# Variation of Two S3b Residues in K_V_4.1–4.3 Channels Underlies Their Different Modulations by Spider Toxin κ-LhTx-1

**DOI:** 10.3389/fphar.2021.692076

**Published:** 2021-06-10

**Authors:** Zhen Xiao, Piao Zhao, Xiangyue Wu, Xiangjin Kong, Ruiwen Wang, Songping Liang, Cheng Tang, Zhonghua Liu

**Affiliations:** The National and Local Joint Engineering Laboratory of Animal Peptide Drug Development, College of Life Sciences, Hunan Normal University, Changsha, China

**Keywords:** K_V_4 channels, spider toxin, voltage-dependent inhibition, molecular basis, anti-arrhythmic drugs

## Abstract

The naturally occurred peptide toxins from animal venoms are valuable pharmacological tools in exploring the structure-function relationships of ion channels. Herein we have identified the peptide toxin κ-LhTx-1 from the venom of spider *Pandercetes sp* (the Lichen huntsman spider) as a novel selective antagonist of the K_V_4 family potassium channels. κ-LhTx-1 is a gating-modifier toxin impeded K_V_4 channels’ voltage sensor activation, and mutation analysis has confirmed its binding site on channels’ S3b region. Interestingly, κ-LhTx-1 differently modulated the gating of K_V_4 channels, as revealed by toxin inhibiting K_V_4.2/4.3 with much more stronger voltage-dependence than that for K_V_4.1. We proposed that κ-LhTx-1 trapped the voltage sensor of K_V_4.1 in a much more stable resting state than that for K_V_4.2/4.3 and further explored the underlying mechanism. Swapping the non-conserved S3b segments between K_V_4.1(_280_FVPK_283_) and K_V_4.3(_275_VMTN_278_) fully reversed their voltage-dependence phenotypes in inhibition by κ-LhTx-1, and intensive mutation analysis has identified P282 in K_V_4.1, D281 in K_V_4.2 and N278 in K_V_4.3 being the key residues. Furthermore, the last two residues in this segment of each K_V_4 channel (P282/K283 in K_V_4.1, T280/D281 in K_V_4.2 and T277/N278 in K_V_4.3) likely worked synergistically as revealed by our combinatorial mutations analysis. The present study has clarified the molecular basis in K_V_4 channels for their different modulations by κ-LhTx-1, which have advanced our understanding on K_V_4 channels’ structure features. Moreover, κ-LhTx-1 might be useful in developing anti-arrhythmic drugs given its high affinity, high selectivity and unique action mode in interacting with the K_V_4.2/4.3 channels.

## Introduction

The voltage-gated potassium channels (K_V_s) are the molecular basis of K^+^ outflow from the cells in response to membrane depolarizations. Among them, the K_V_4 (*Shal*) family which contains three members as K_V_4.1, K_V_4.2, and K_V_4.3 is mostly featured by being activated at sub-threshold membrane potentials and possessing rapid activation/inactivation kinetics (Baldwin et al., 1991; Serôdio et al., 1994; Serôdio et al., 1996; Birnbaum et al., 2004). Each K_V_4 channel is constructed by symmetrical assembling of four pore-forming K_V_4. x subunits (K_V_4.1, K_V_4.2, and K_V_4.3 subunit encoded by the KCND1, KCND2, and KCND3 gene, respectively), in which a subunit is composed of six transmembrane segments (S1–S6), with S1–4 constructing the voltage sensor domain (VSD) and S5–6 forming the pore domain (PD). Meanwhile, several auxiliary subunits are associated with K_V_4 channels to profoundly modulate their gating and membrane trafficking, including K_V_β, KChIPs, DPPX, and so on (An et al., 2000; Yang et al., 2001; Nadal et al., 2003). As a subset of the K_V_s superfamily, the K_V_4 channels share a general gating mode with all other K_V_s: driven by membrane depolarizaitons, the S4 segment which harbors regularly distributed R or K residues on it moves outward in the gating pore formed by S1–3, such conformation change in VSD is then transduced to PD to trigger pore opening (Lu et al., 2002; Barghaan and Bähring, 2009). In heart, K_V_4.2, and K_V_4.3 channels are the molecular basis of I_to_ currents, which affects calcium inflow and myocardial contractility (Nerbonne and Kass, 2005; Niwa and Nerbonne, 2010). While in neurons, K_V_4.2, and K_V_4.3 channels are responsible for the I_A_ currents that regulates neuron excitability (Kim and Hoffman, 2008; Carrasquillo and Nerbonne, 2014). Given the crucial role of K_V_4 channels in physiological conditions, their dysfunctions in heart are associated with diseases like Brugada syndrome, atrial fibrillation, hypertrophy and heart failure (Giudicessi et al., 2011; Olesen et al., 2013; Yang and Nerbonne, 2016; Drabkin et al., 2018). Moreover, genetic studies have identified mutations in K_V_4.2 and K_V_4.3 channels causing autism, epilepsy, and spinocerebellar ataxia type in central nervous system (CNS) (Duarri et al., 2012; Smets et al., 2015; Lin et al., 2018). Besides, reduced expression of K_V_4 channels in peripheral neurons is also closely related with chronic pain conditions (Chien et al., 2007; Zemel et al., 2018). Therefore, regulating the activity of Kv4 channels represents a promising strategy for diseases treatment, and pharmacological agents acting on K_V_4 channels are valuable drug candidates (Feng et al., 1997; Ma et al., 2015; Zhang et al., 2019a).

Animal venoms are rich in peptide toxins acting on various types of ion channels and receptors. Up to date, lots of venom-derived peptide antagonists for the K_V_4 channels have been characterized (Swartz and MacKinnon, 1995; Sanguinetti et al., 1997; Escoubas et al., 2002; Yuan et al., 2007; Bougis and Martin-Eauclaire, 2015; Zhang et al., 2019b). They could be roughly classified into two groups as pore blockers and gating modifiers based on their action modes. Scorpion toxins including Aa1, AaTX1/2, BmTX3, AmmTX3, and Discrepin in the α-KTX15 family are classical pore blockers of K_V_4 channels, which function by binding to and physically occluding the K^+^ conductive pathway (Pisciotta et al., 2000; Vacher et al., 2001; D'Suze et al., 2004; Maffie et al., 2013; Mlayah-Bellalouna et al., 2014). A critical lysine or arginine residue is commonly identified in these toxins, which uses its side chain to compete the K^+^ binding site in the pore. The pore geography of channel might be important for these toxins’ binding as well. For example, AmmTX3 inhibits less K_V_4 currents in heterologous expression system than that in native neurons, which might be caused by the presence of auxiliary subunits DPP6/10 in native tissue but not in cultured cell lines helps to rearrange the structure of the channel pore, allowing for a better residence of toxin in it (Maffie et al., 2013). On the other hand, lots of spider toxins were characterized as gating modifiers which inhibit K_V_4 channels’ currents by trapping their voltage sensor in a resting state, hindering its activation in response to membrane depolarizations, such as Heteropodatoxins (HpTx1-3), JZTX-V, JZTX-XII, HaTx1, and ScTx (Swartz and MacKinnon, 1995; Sanguinetti et al., 1997; Escoubas et al., 2002; Zarayskiy et al., 2005; Yuan et al., 2007; Zhang et al., 2019b). Based on the action mechanism, these toxins would usually shift the voltage-dependent activation and/or inactivation kinetics of the channel. Interestingly, some toxins could even act on different K_V_ subtypes by different mechanisms, as exemplified by Ctri9577 isolated from the venom of the scorpion *Chaerilus tricostatus*, which is recognized as a pore blocker of the K_V_1.3 channel, but as a gating modifier of the K_V_4.3 channel (Xie et al., 2014). Among the K_V_4 gating modifier toxins, the action mechanism of HpTx2 was intensively studied. This toxin is isolated from the venom of the spider *Heteropoda venatoria* and is shown to bind to the same S3b region in K_V_4.1 and K_V_4.3. Moreover, although HpTx-2 uses a general common mechanism to inhibit K_V_4.1 and K_V_4.3 as hindering their voltage sensor activation, it inhibits these two channels with different voltage-dependence, as revealed by HpTx-2 inhibiting significantly more K_V_4.3 currents than that for K_V_4.1 at 0 mV but essentially the same proportion at a much more stronger depolarization of +50 mV, resulting in a larger G-V shift in K_V_4.3. Swapping the non-conserved S3b segments between K_V_4.1 and K_V_4.3 has switched their voltage-dependence phenotypes (i.e., less voltage-dependence in K_V_4.1 vs. large voltage-dependence in K_V_4.3). The Markov model used to interpret these data showed HpTx2 mostly affected channel’s voltage-dependent closed states transition from C_0_ to C_4_ in K_V_4.3, but the voltage-independent pre-open to open states transition (C_4_→O transition) in K_V_4.1 (DeSimone et al., 2009; DeSimone et al., 2011). Despite the recent advances, the molecular mechanisms of peptide toxins acting on K_V_4 channels are far to be elucidated. Identifying novel antagonists of K_V_4 channels and investigating their action mechanisms will certainly deepen our understanding on channels’ gating and structure-function relationships.

In the present study, we have purified and characterized the peptide toxin, κ-LhTx-1, as a novel selective antagonist of the K_V_4.1–4.3 channels. Notably, this toxin inhibited K_V_4.2 and K_V_4.3 currents with stronger voltage-dependence than that in K_V_4.1. κ-LhTx-1 shifted the G-V curve of K_V_4.1 to the depolarizing direction to a much bigger extent than that in K_V_4.2/4.3, which is distinct from the effect of HpTx-2. We proposed that κ-LhTx-1 trapped the K_V_4.1 voltage sensor in a more stable resting state than that for the K_V_4.2/4.3 channels. Mechanism studies revealed that although κ-LhTx-1 binds to the same S3b region in K_V_4.1–4.3 channels, two residues variation in the S3b region made their gating be differently modulated by κ-LhTx-1. These data advanced our understanding on K_V_4 channels’ structure features, besides, κ-LhTx-1 might be useful in developing anti-arrhythmic drugs.

## Material and Methods

### Venom and Toxin Purification

Spiders *Pandercetes sp* were captured in Guangxi Province in China and maintained in our laboratory for short time, fed weekly with mealworms and water. The venom was collected by an electrical stimulation method, lyophilized and preserved at −80°C. The crude venom was dissolved in ddH_2_O to a final concentration of 5 mg/ml and immediately subjected to the first round of semi-preparative RP-HPLC purification (C18 column, 10 × 250 mm, 5 μm, Welch Materials Inc., Shanghai, China) using a 55-min linear acetonitrile gradient from 5 to 60% at 3 ml/min flow rate (Hanbon HPLC system equipped with NP7000 serials pump and NU3000 serials UV/VIS detector, Hanbon Sci. and Tech. Huai’an, China). The fraction containing κ-LhTx-1 was collected, lyophilized, and subjected to the second round of analytical RP-HPLC purification (C18 column, 4.6 × 250 mm, 5 μm, Welch Materials Inc., Shanghai, China) using a 35-min linear acetonitrile gradient from 25 to 42.5% at 1 ml/min flow rate (Waters 2695 HPLC system, Waters Corporation, Milford, MA, United States). The purity and molecular weight (MW) of the toxin was analyzed by MALDI-TOF mass spectrometry (AB SCIEX TOF/TOF™ 5800 system, Applied Biosystems, Foster City, CA, United States). All mass spectra were acquired in the positive reflectron mode, and the laser intensity was set to 4,000. The matrix for mass spectrometry analysis was α-Cyano-4-hydroxycinnamic acid (CCA).

### Toxin Characterization

κ-LhTx-1’s N-terminal sequence of 10 residues long was determined by Edman degradation using an automatic protein sequencer (SHIMADZU PPSQ31A, Kyoto, Japan). Its full sequence was determined by blasting the N-terminal sequence against our local peptides sequence database derived from the venom gland cDNA library of *Pandercetes sp* (unpublished data). The identity of the toxin was cross-checked by matching its experimentally determined MW with the MW derived from the hit sequence.

### Solid-phase Synthesis and In-Vitro Refolding of κ-LhTx-1

κ-LhTx-1 linear peptide was synthesized using a Fmoc [N-(9-fluorenyl)methoxycarbonyl]/tert-butyl strategy and HOBt/TBTU/NMM coupling method. The produced linear peptide was poorly dissolved in the basic refolding solution [5 mM GSH, 0.5 mM GSSG, 100 mM NaCl, 0.1 M Tris-HCl (pH = 7.4)], therefore 4 M guanidine hydrochloride was added to improve its solubility. Peptide was refolded at a concentration of 0.1 mg/ml, after 5 h stir at 4°C, the guanidine hydrochloride concentration in the refolding mix was sequentially diluted to 3, 2, and 1 M with basic refolding solution (one dilution per 5 h). RP-HPLC and MALDI-TOF MS analysis was used to monitor the refolding process. At last, the refolding reaction was terminated by adding TFA to a final concentration of 0.2%, and the mix was subjected to RP-HPLC purification to collect the correctly refolded toxin (C18 column, 10 × 250 mm, 5 μm, Welch Materials Inc., Shanghai, China; a 45-min linear acetonitrile gradient from 5 to 50% was used, the flow rate is 3 ml/min). The correct refolding of synthesized κ-LhTx-1 was also confirmed by its co-elution with the native κ-LhTx-1 in RP-HPLC analysis (C18 column, 4.6 × 250 mm, 5 μm, Welch Materials Inc., Shanghai, China) using a 50-min linear acetonitrile gradient from 5 to 55% at 1 ml/min flow rate (Waters 2795 HPLC system, Waters Corporation, Milford, MA, United States).

### Plasmids, Site-Directed Mutation, Cell Culture and Transient Transfection

The cDNA of hK_V_1.1, hK_V_1.3, rK_V_1.4, hK_V_1.5, rK_V_2.1, hK_V_3.1–3.4, mK_V_4.1, rK_V_4.2, and rK_V_4.3 were subcloned in the eukaryotic expression vector pCDNA3.1 or pCMV-blank. Channel mutants were made by a site-directed mutation method. Briefly, a pair of oppositely directed primers with 15 bp overlap at their 5′ ends and the designed mutation site were used to amplify the parental channel plasmid, then the PCR mix was treated with DpnI to remove the template. 10 μL digestion mix was directly used to transform 100 μL DH5α chemical competent cells. The correct mutation made by this procedure was finally confirmed by DNA sequencing. CHO-K1 cells (ATCC^®^ CCL-61™) were grown in DMEM-F12 mixed medium (1:1) supplemented with 10% FBS and maintained in standard conditions (saturated humidity, 37°C, 5% CO_2_). Channel plasmid was co-transfected with pEGFP-N1 (encodes the green fluoresence protein) into CHO-K1 cells using lipofectamine 2000 following the manufacturer’s instructions. 4–6 h after transfection, cells were seeded onto poly-lysine (PLL) coated coverslips, and 24–36 h later, transfected cells were ready for patch-clamp analysis. It should be note that K_V_4.2 and its mutants were also co-expressed with hKChIP1 to promote their functional expressions.

### Whole-Cell Patch Clamp Recording

Whole-cell patch clamp recording was performed in an electrophysiology platform equipped with MultiClamp 700B amplifier and Axon Digidata 1550 AD/DA convertor (Axon Instruments, Irvine, CA, United States). Data were acquired using the pClamp software (Axon Instruments, Irvine, CA, United States). All experiments were performed at room temperature. The bath solution contains (in mM): 140 NaCl, 5 KCl, 1 MgCl_2_, 2 CaCl_2_, 10 glucose, and 10 HEPES (pH = 7.3). The pipette solution contains (in mM): 140 KCl, 2.5 MgCl_2_, 11 EGTA, and 10 HEPES (pH = 7.3). Series resistance was kept below 10 MΩ and compensated to 80%. The concentration–response curves were fitted by a Hill logistic equation to estimate the potency (IC_50_) of the toxin. The whole cell conductance (G) at each depolarizing voltage (V) was determined using the equation: G = I/(V−V_rev_), where I and V_rev_ represents the current amplitude and the reversal potential, respectively. In the present study, V_rev_ for K^+^ current is determined to be −85.61 mV using the Nernst equation. G-V curve was obtained by plotting the normalized G as a function of V and fitted by the Boltzmann equation: y = 1/{1 + exp [(V_a_−V)/K]}, in which V_a_, V, and K represents half-maximum activation voltage, test voltage and slope factor, respectively. The steady-state fast inactivation of K_V_4 channels was measured using a classical two-pulses protocol: cell was held at −120 mV, and a train of conditional voltages (−120–40 mV, in 10 mV increment, 1,000 ms) were applied to induce channel inactivation, followed by a +60 mV test pulse (300 ms) to assess the proportion of non-inactivated channels; the sweep interval was set to 10 s. Currents at the test pulse (I) were normalized to the maximum value (I_max_) and plotted as a function of the conditional voltage (V), the curve was fitted by the Boltzmann equation: I/I_max_ = A + (1−A)/{1 + exp [(V−V_h_)/K]}, where V_h_ is the half-maximum inactivation voltage, A represents the minimum channel availability, and K is the slope factor. Gating currents of K_V_4.1–4.3 channels were measured as previously reported (Tilley et al., 2019). Briefly, the ionic pore currents were abolished by replacing K^+^ with NMDG^+^ in the pipette solution, and 10 µM Cs^+^ was present in the bath solution to occupy the selectivity filter of K_V_ channels to prevent decay of the gating currents during recording.

### Data Analysis

Data were presented as MEAN ± SEM, n represents the number of separate experimental cells. Data were analyzed using the software Clampfit 10.5 (Axon Instruments, Irvine, CA, United States), Graphpad Prism 5.01 (GraphPad Software, La Jolla, CA, United States) and Excel 2010 (Microsoft Corporation, Redmond, WA, United States). Statistical significance was assessed using ONE-WAY ANOVA, and significant difference was accepted at *p* < 0.05.

## Results

### κ-LhTx-1 Is a Novel K_V_4.1 Channel Antagonist

**FIGURE 1:**
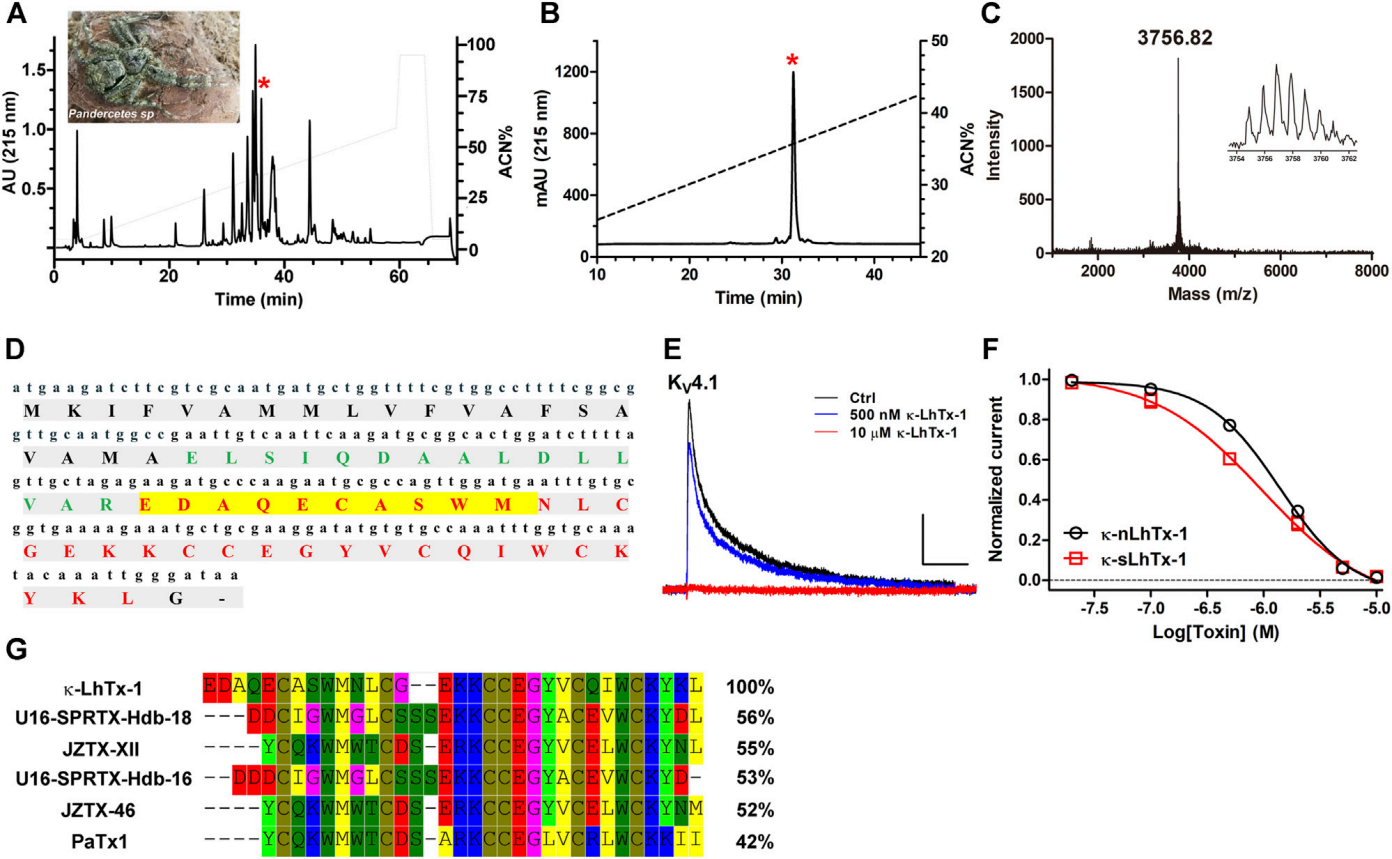
Purification and characterization of κ-LhTx-1. **(A)**, RP-HPLC profile of *Pandercetes sp* (inset) *venom*, the red asterisk labeled peak contains κ-LhTx-1. **(B)**, κ-LhTx-1 was purified to homogeneity by analytical RP-HPLC (red asterisk labeled peak). **(C)**, MALDI-TOF MS analysis of purified κ-LhTx-1, the inset shows its single isotopic molecular weight. **(D)**, cDNA and protein sequence of κ-LhTx-1. The signal peptide, propeptide and mature peptide was shown in black bold, green bold and red bold, respectively. N-terminal sequence of κ-LhTx-1 was determined by Edman degradation and highlighted in yellow. **(E)**, Representative traces showing K_V_4.1 was concentration-dependently inhibited by κ-nLhTx-1 (n = 5). Currents were elicited by a 300 ms depolarization to +30 mV from −80 mV holding. Scale bars, 0.5 nA × 50 ms. **(F)**, The concentration-response curves of κ-nLhTx-1 and κ-sLhTx-1 inhibiting K_V_4.1 at + 30 mV, the IC_50_ values were determined as 1.36 ± 0.38 μM and 0.87 ± 0.15 μM for κ-nLhTx-1 and κ-sLhTx-1, respectively (n = 5). **(G)**, Sequence alignment of κ-LhTx-1 with several toxins in the database using MEGA8.0.

The venom components of the spider *Pandercetes sp* (the Lichen huntsman spider; inset in Figure 1A) are largely unexplored to date. In an effort to characterize the peptide toxins in its venom and map their activities on various ion channels, we identified a RP-HPLC purified fraction of the venom with potent inhibitory effect on the Kv4.1 channel. The RP-HPLC retention time of this active fraction (star labeled peak) is 36.4 min (Figure 1A). Furthermore, the second round of analytic RP-HPLC purification showed that this component was readily purified to homogeneity (Figure 1B). Its purity was also confirmed by MALDI-TOF MS analysis and the molecular weight was determined as 3,756.82 Da (M + H^+^), supporting that this K_V_4.1 active component is a peptide toxin (Figure 1C). By combining Edman degradation sequencing and venom gland cDNA library analysis (unpublished data), we determined this toxin’s full amino acid sequence and named it as κ-LhTx-1 (Figure 1D), following the nomenclature rules proposed by King, G.F et al. (King et al., 2008). Blasting κ-LhTx-1 sequence in public database showed that it has medium sequence identity with U6-SPRTX-Hdb-18 (56%) and U6-SPRTX-Hdb-16 (53%) from the venom of spider *Heteropoda davidbowie*, JZTx-XII (55%) and JZTx-46 (52%) from the venom of spider *Chilobrachys guangxiensis* (Figure 1G). Besides, it showed 42% sequence homology to the potent K_V_4 channels antagonist PaTx1 (Diochot et al., 1999) (Figure 1G). κ-LhTx-1 concentration-dependently inhibited the peak current of K_V_4.1 channel with an IC_50_ 1.36 ± 0.38 μM at +30 mV (Figure 1E and black curve in Figure 1F). To further confirm the activity of κ-LhTx-1, we produced linear κ-LhTx-1 by solid-phase synthesis and reconstructed its native disulfide bonds by *in vitro* refolding. The synthetic product was eluted as a major peak at 39 min in RP-HPLC purification (Supplementary Figure S1A), and MALDI-TOF MS analysis showed it contains two peptides, with MW of 3,764.12 Da corresponding to linear κ-LhTx-1 and MW of 2,523.47 Da representing a byproduct (Supplementary Figure S1B). This fraction was collected and directly subjected to the refolding process. Using guanidine hydrochloride to assist κ-LhTx-1 refolding (see Materials and Methods), we finally get approximately 10% of the linear peptide correctly refolded (Supplementary Figures S1C–1F). We referred to the synthetic and native κ-LhTx-1 as κ-sLhTx-1 and κ-nLhTx-1, respectively. The MWs of κ-sLhTx-1 and κ-nLhTx-1 match well (Supplementary Figure S1G and Figure 1C), RP-HPLC analysis also showed they were co-eluted as a single peak (Supplementary Figure S1H). Furthermore, κ-sLhTx-1 inhibited the peak current of K_V_4.1 channel with an IC_50_ of 0.87 ± 0.15 μM at +30 mV (red curve in Figure 1F), which is not significantly different from that of κ-nLhTx-1. These data clearly confirmed the activity of κ-LhTx-1 on KV4.1. Except the initial screening experiments, we used the synthesized toxin throughout this study, and κ-sLhTx-1 was written as κ-LhTx-1 for clarity hereafter.

### κ-LhTx-1 Differently Modulates the Gating of K_V_4 Channels

**FIGURE 2:**
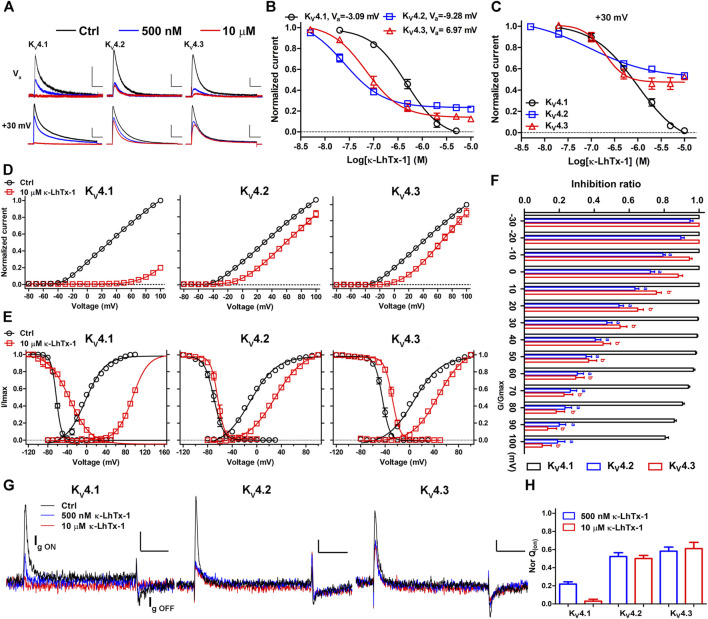
κ-LhTx-1 inhibits K_V_4 channels with different voltage-dependence. **(A)**, Representative traces showing the inhibition of K_V_4 channels by κ-LhTx-1 at different depolarizing voltages (Upper panel: test at V_a_ voltage for K_V_4.1, K_V_4.2 and K_V_4.3, respectively; Lower panel: test at +30 mV; n = 5–6). Holding potential was set to -80 mV, the V_a_ value for each K_V_4 subtype was as shown in **(B)**. Scale bars, 0.5 nA × 50 ms (Upper panel), 2 nA × 50 ms (Lower panel). **(B, C)**, The concentration-response curves of κ-LhTx-1 inhibiting K_V_4 channels at their V_a_ depolarization voltages **(B)** or +30 mV **(C)**. The IC_50_ values in K_V_4.1, K_V_4.2 and K_V_4.3 were determined as 0.51 ± 0.11 μM and 0.87 ± 0.15 μM, 0.03 ± 0.01 μM and 0.14 ± 0.05 μM, 0.06 ± 0.02 μM and 0.16 ± 0.04 μM, at V_a_ voltage and +30 mV, respectively (n = 5–6). **(D)**, The I-V relationships of K_V_4.1, K_V_4.2 and K_V_4.3 channels before (black) and after (red) 10 μM κ-LhTx-1 treatment, currents at all voltages were normalized to the control current (before toxin treatment) at +100 mV in each group (n = 5–8). **(E)**, The steady-state activation/inactivation curves of K_V_4.1, K_V_4.2 and K_V_4.3 channels before (black) and after (red) 10 μM κ-LhTx-1 treatment (n = 5–10). **(F)**, The inhibition ratio of 10 µM κ-LhTx-1 on the currents mediated by K_V_4.1 (black), K_V_4.2 (blue) and K_V_4.3 (red) at different depolarizing voltages (n = 5–8). a, *p* < 0.05 when comparing the ratio in K_V_4.2 with that in K_V_4.1; b, *p* < 0.05 when comparing the ratio in K_V_4.3 with that in K_V_4.1(ONE-WAY ANOVA). **(G)**, Representative traces showing κ-LhTx-1 inhibited the gating currents of K_V_4.1–4.3 channels. Cells were held at -100 mV, currents were elicited by a 20 ms depolarization to +30 mV, note the ON gating current (I_g ON_) was larger than the OFF gating current (I_g_ OFF) due to gating charge immobilization; Scale bar: 100 pA × 5 ms; n = 5–6. **(H)**, Statistics of inhibition of gating charge (Q; integral of I_g_ ON) movement by application of 500 nM and 10 µM κ-LhTx-1, note 500 nM and 10 µM toxin inhibited essentially the same proportion of gating charge movement in K_V_4.2/4.3 channels but not in K_V_4.1 (n = 5–6).

An expanded survey of κ-LhTx-1 activity on several other K_V_ channels showed that it did not remarkably affect the currents of K_V_1.1, K_V_1.3–1.5, K_V_2.1 and K_V_3.1–3.4 channels even at 10 µM concentration (Supplementary Figures S2A–I). However, as it for K_V_4.1, κ-LhTx-1 also potently inhibited the currents of K_V_4.2 and K_V_4.3 channels, all in a reversible manner (Figures 2A–C and Supplementary Figures S2J–L). This is not surprising due to the extremely high homology between these K_V_4 family members. At V_a_ depolarizing voltage of K_V_4.1, K_V_4.2, and K_V_4.3, κ-LhTx-1 almost fully inhibited their currents with an IC_50_ of 0.51 ± 0.11, 0.03 ± 0.01, and 0.06 ± 0.02 μM, respectively, showing relatively higher potency against K_V_4.2 and K_V_4.3 than that for K_V_4.1 (Upper panel in Figure 2A,B). However, when testing its activity at the same depolarizing voltage of +30 mV, κ-LhTx-1 at the saturating dose of 10 μM can only fully inhibit K_V_4.1 currents but not the other two channels, with a maximum inhibition ratio of 53.5 ± 2.2% for K_V_4.2 and 47.5 ± 1.5% for K_V_4.3 (Lower panel in Figure 2A,C). The apparent IC_50_ value at +30 mV was determined as 0.87 ± 0.15, 0.14 ± 0.05, and 0.16 ± 0.04 μM for K_V_4.1, K_V_4.2, and K_V_4.3, respectively (Figure 2C). The loss of κ-LhTx-1’s potency on K_V_4.2 and K_V_4.3 at a stronger depolarization of +30 mV was not caused by reduced toxin binding (reduced affinity) as the toxin’s inhibitory effect already reached the platform (Figure 2C). We reasoned that the voltage-dependent inhibition of κ-LhTx-1 on the K_V_4.2 and K_V_4.3 channels might be the underlying mechanism. Therefore, we analyzed the effect of κ-LhTx-1 on the I-V relationships of K_V_4 channels. It should be note that we saturated channels on cell membrane with toxin by using 10 µM κ-LhTx-1 treatment, which made at least one subunit of each channel is bound with a toxin molecule, allowing us to compare toxin’s effects between different K_V_4 channels. As shown in Figure 2D, toxin treatment all right-forwardly shifted the I-V relationships of three K_V_4 channels, but to distinct extents. In K_V_4.1, toxin fully inhibited the currents at voltages below +60 mV, and only depolarizations stronger than +70 mV could partially reopen the toxin-bound channels, which caused a very large shift of the I-V relationship. In contrast, only a small shift was observed in K_V_4.2 and K_V_4.3. We measured the inhibition ratio of κ-LhTx-1 on three K_V_4 channels at each depolarizing voltage, which showed that the toxin’s inhibition decreased quickly with the increment of the depolarizing voltage in K_V_4.2 and K_V_4.3, while it was affected by voltage to a much less extent in K_V_4.1 (Figure 2F). The differences between K_V_4.1 and K_V_4.2/4.3 channels were more pronounced at higher depolarizing voltages (Figure 2F). Consistent with the right-forwardly shifted I-V relationships, the G-V relationships of the three K_V_4 channels were profoundly changed by toxin. 10 μM κ-LhTx-1 shifted the V_a_ of K_V_4.1, K_V_4.2, and K_V_4.3 channel by 98.28 ± 2.66, 39.52 ± 2.55, and 39.88 ± 2.19 mV, respectively (Figure 2E and Supplementary Table S1). Similar effects on channels’ steady-state inactivation were also observed, with 10 μM toxin shifting the V_h_ of K_V_4.1, K_V_4.2, and K_V_4.3 channel by 29.27 ± 2.05, 6.25 ± 2.45, and 14.83 ± 3.34 mV, respectively (Figure 2E and Supplementary Table S1). These data strongly implied that κ-LhTx-1 acted on K_V_4 channels as a gating modifier stabilizing the deactivated voltage sensors, which was directly validated by that the toxin inhibited their gating currents (Figures 2G,H). More importantly, κ-LhTx-1 inhibited K_V_4.2/4.3 channels with much more stronger voltage-dependence than that in K_V_4.1 (i.e., K_V_4.1 and K_V_4.2/4.3 channels had different voltage-dependence phenotypes in inhibition by κ-LhTx-1), suggesting the gating of three K_V_4 channels was differently modulated by toxin.

### The Non-conserved S3b Segments in K_V_4 Channels Determine Their Different Modulations by κ-LhTx-1

**FIGURE 3:**
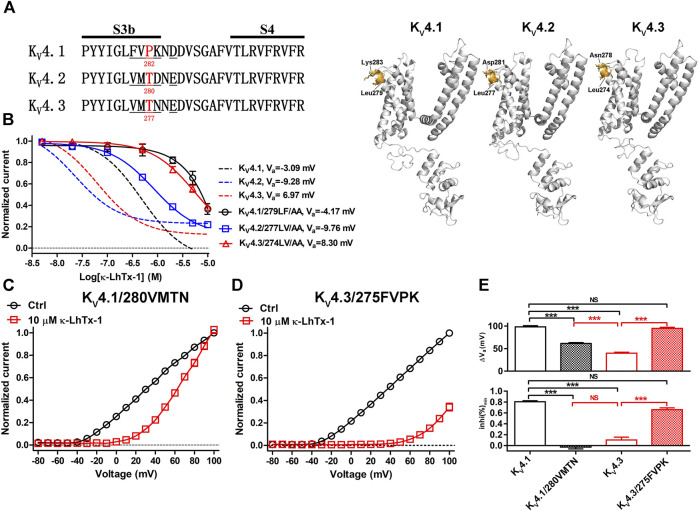
Characterizing the non-conserved S3b segments in K_V_4.1 and K_V_4.3 as the key molecular determinants. **(A)**, Left: sequence alignment of the K_V_4 channels’ S3b-S4 segments, the non-conserved S3b regions are underlined, and the number below the residue indicates its location in the sequence (in mK_V_4.1, rK_V_4.2 and rK_V_4.3 numbering); Right: Locations of the non-conserved S3b segments in the simulated structures of K_V_4.1–4.3 channels as determined by SWISS-MODEL using 5WIE (PDB ID) as the template (https://swissmodel.expasy.org/), note only one subunit for each channel was shown for clarity. **(B)**, The concentration-response curves of κ-LhTx-1 inhibiting the K_V_4.1/279LF/AA, K_V_4.2/277LV/AA and K_V_4.3/274LV/AA mutants at their respective V_a_ depolarizing voltages (n = 5–7). For comparison, the curves for wild-type K_V_4.1, K_V_4.2 and K_V_4.3 were shown in dashed lines. K_V_4.1/279LF/AA, K_V_4.2/277LV/AA and K_V_4.3/274LV/AA were made by mutating L279/F280 in K_V_4.1, L277/V278 in K_V_4.2 and L274/V275 in K_V_4.3, to alanines, respectively. **(C)** and **(D)**, The I-V curve of K_V_4.1/280VMTN **(C)** and K_V_4.3/275FVPK **(D)** before (black) and after (red) 10 μM κ-LhTx-1 treatment, currents at all voltages were normalized to the control current (before toxin treatment) at +100 mV in each group (n = 5). K_V_4.1/280VMTN and K_V_4.3/275FVPK chimeras were made by replacing the _280_FVPK_283_ segment in K_V_4.1 with VMTN and the _275_VMTN_278_ segment in K_V_4.3 with FVPK, respectively. **(E)**, The statistical diagram of the ∆V_a_ and inhi%_(min)_ values for wild-type K_V_4.1, K_V_4.3, K_V_4.1/280VMTN and K_V_4.3/275FVPK, showing swapping the non-conserved S3b segments between K_V_4.1 and K_V_4.3 exchanged their voltage-dependence phenotypes in inhibition by κ-LhTx-1 (***, *p* < 0.001; NS, not significantly different; ONE-WAY ANOVA; n = 5–13).

We then explored the structure determinants in K_V_4 channels underlying their different modulations by κ-LhTx-1 using a chimeric channel strategy. The toxin has the minimum inhibitory effect on K_V_4 channels at +100 mV, we used both the V_a_ shift (termed as ∆V_a_) and the inhibition ratio at +100 mV (termed as inhi%_(min)_) to quantitatively evaluate the voltage-dependence of toxin inhibiting them, with smaller ∆V_a_ and inhi%_(min)_ values representing stronger voltage-dependence. This strategy was justified as we observed a mutant (K_V_4.3/T277P) with unchanged ∆V_a_ but its voltage-dependent inhibition by toxin was really attenuated, as reflected by the significantly increased inhi%_(min)_ value. In most cases, however, these two values would decrease or increase concomitantly. The different modulations of κ-LhTx-1 on K_V_4 channels does not raise from toxin binding with different regions on them, as mutation analysis showed that _279_LF/AA mutations in K_V_4.1, as well as its homologous residues mutations, _277_LV/AA in K_V_4.2 and _274_LV/AA in K_V_4.3 (number indicates the location of the mutated segment in sequence), all profoundly weakened the effect of κ-LhTx-1, suggesting κ-LhTx-1 is bound with the same S3b region in K_V_4 channels (Figures 3A,B). Besides, the non-conserved S3b segments neighboring the toxin binding sites in K_V_4 channels (Figure 3A; _280_FVPK in K_V_4.1, _275_VMTN in K_V_4.3, and _278_VMTD in K_V_4.2; number indicates the location of the segment in sequence), were identified as key molecular determinants for the different voltage-dependent modulations of K_V_4.1 and K_V_4.3 by HpTx-2 (DeSimone et al., 2011). We then asked whether they played a similar role in the action of κ-LhTx-1 on K_V_4 channels. K_V_4.1/280VMTN and K_V_4.3/275FVPK chimeric channels were constructed by swapping this S3b segment between K_V_4.1 and K_V_4.3. As a result, toxin treatment caused a much more smaller I-V shift in K_V_4.1/280VMTN than that in K_V_4.1 (Figures 2D, 3C). However, this shift is much more pronounced in K_V_4.3/275FVPK than that in K_V_4.3 (Figures 2D, 3D). In agreement with these observations, the ∆V_a_ value of 61.44 ± 1.98 mV for K_V_4.1/280VMTN and 94.81 ± 2.45 mV for K_V_4.3/275FVPK was significantly different from that for their parental K_V_4.1 (∆V_a_ = 98.28 ± 2.66 mV) and K_V_4.3 (∆V_a_ = 39.88 ± 2.19 mV) channel, respectively (Figure 3E, upper panel). Notably, the ∆V_a_ values for K_V_4.1 and K_V_4.3/275FVPK channels were not significantly different, suggesting that they were modulated by κ-LhTx-1 in the same way (Figure 3E, upper panel). On the other hand, the inhi%_(min)_ value was reduced from 80.58 ± 1.84% in K_V_4.1 to −2.82 ± 3.37% in K_V_4.1/280VMTN, but increased from 10.29 ± 5.13% in K_V_4.3 to 65.91 ± 3.36% in K_V_4.3/275FVPK, which more directly showed an exchange of channel’ voltage-dependence phenotype in inhibition by toxin by swapping this S3b segment between K_V_4.1 and K_V_4.3 (Figure 3E, lower panel). Taken together, these data established that this non-conserved S3b segment containing four residues is the underlying molecular determinant.

### Characterizing the Key Residues in the Non-conserved S3b Segment

**FIGURE 4:**
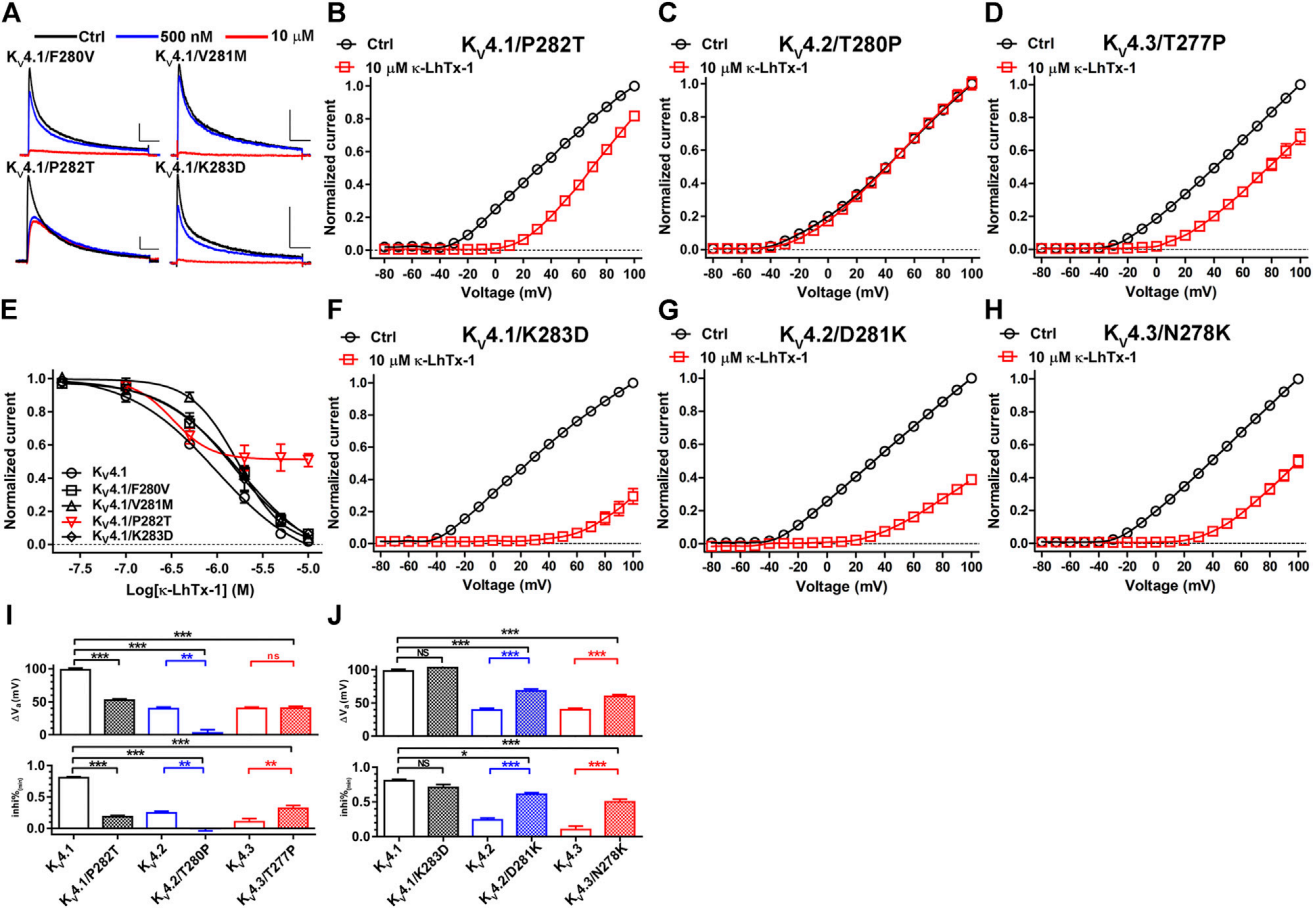
Characterizing the key residues in the non-conserved S3b segment. **(A)**, Representative traces of κ-LhTx-1 inhibiting K_V_4.1/F280V, K_V_4.1/V281M, K_V_4.1/P282T and Kv4.1/K283D mutants (n = 5). **(B–D)**, The I-V curves of K_V_4.1/P282T **(B)**, K_V_4.2/T280P **(C)** and K_V_4.3/T277P **(D)** mutants before (black) and after (red) 10 μM κ-LhTx-1 treatment (n = 5–6). Currents at all voltages were normalized to the control current (before toxin treatment) at +100 mV in each group. **(E)**, The concentration-response curves of κ-LhTx-1 inhibiting K_V_4.1/F280V, K_V_4.1/V281M, K_V_4.1/P282T and K_V_4.1/K283D mutants at +30 mV (n = 5). The curve for K_V_4.1 was also included for comparison. **(F–H)**, The I-V curves of K_V_4.1/K283D **(F)**, K_V_4.2/D281K **(G)** and K_V_4.3/N278K **(H)** mutants before (black) and after (red) 10 μM κ-LhTx-1 treatment (n = 5–6). Currents at all voltages were normalized to the control current (before toxin treatment) at +100 mV in each group. **(I)** and **(J)**, The statistical diagram of the ∆V_a_ and inhi%_(min)_ values of wild-type K_V_4 channels and mutants referred in this Figure, showing that P282T mutation in K_V_4.1, D281K mutation in K_V_4.2, as well as N278K mutation in K_V_4.3 dramatically changed channel’s voltage-dependence phenotype in inhibition by κ-LhTx-1 (*, *p* < 0.05; **, *p* < 0.01; ***, *p* < 0.001; ns, not significantly different; ONE-WAY ANOVA; n = 5–8).

To characterize the key residues in this non-conserved S3b segment determining the voltage-dependence phenotypes, we firstly mutated each of the four residues in K_V_4.1 to its counterpart in K_V_4.2 and tested toxin’s inhibition on them. At +30 mV, F280V, V281M and K283D mutations in K_V_4.1 only slightly reduced κ-LhTx-1 potency (Figures 4A,E). The K_V_4.1/P282T mutant, however, was not fully inhibited even after 10 µM toxin treatment, implying its voltage-dependent inhibition by toxin might be changed (Figures 4A,E). Actually, toxin treatment only moderately shifted its I-V relationship, which is distinct from that observed in wild-type K_V_4.1 (Figures 2D, 4B). The ∆V_a_ and inhi%_(min)_ values for K_V_4.1/P282T were significantly reduced compared with those for K_V_4.1 but were close to those for K_V_4.2/K_V_4.3 channels (Figure 4I), implying K_V_4.1/P282T is inhibited by κ-LhTx-1 with strong voltage-dependence as K_V_4.2 and K_V_4.3. To our surprising, its reverse mutation in K_V_4.2 (K_V_4.2/T280P) made the channel resistant to toxin inhibition at all voltages (Figures 4C,I). It might be caused by toxin binding with this mutant channel in a silent manner, i.e., toxin binding does not remarkably interfere with channel activation. In line with the observations in K_V_4.1/P282T, the T277P mutation in K_V_4.3 (K_V_4.3/T277P) partially restored the voltage-dependence phenotype of K_V_4.1, as revealed by a significantly elevated inhi%_(min)_ value compared with that of wild-type K_V_4.3 (Figures 4D,I, lower panel). Nonetheless, its ∆V_a_ value was not significantly changed (Figure 4I, upper panel). Therefore, the K_V_4.1/P282T data argued a critical role of P282 in K_V_4.1, but the observations in K_V_4.2/T280P and K_V_4.3/T277P suggested the possible involvement of other key residue.

Another typical divergent between the non-conserved S3b segments of K_V_4 channels is the charge state of the last residue, with a positively charged K283 in K_V_4.1, a negatively charged D281 in K_V_4.2, and a neutralized N278 in K_V_4.3 (Figure 3A). We asked whether this difference also contributed to determine their distinct voltage-dependence phenotypes in inhibition by κ-LhTx-1. In consistent with the data in Figures 4A,E, K283D mutation in K_V_4.1 did not remarkably change channel’s voltage-dependence phenotype in inhibition by κ-LhTx-1, as revealed by a greatly shifted I-V relationship after toxin treatment (Figure 4F), as well as unchanged ∆V_a_ and inhi%_(min)_ compared with wild-type K_V_4.1 (Figure 4J). Contrarily, the D281K mutation in K_V_4.2, as well as the N278K mutation in K_V_4.3 greatly restored the voltage-dependence phenotype of K_V_4.1, with toxin treatment greatly shifting the I-V relationships of these two mutant channels (Figures 4G,H). Analyzing the ∆V_a_ and inhi%_(min)_ values showed the toxin inhibited K_V_4.2/D281K and K_V_4.3/N278K mutants with much less voltage-dependence than that in their parental K_V_4.2 and K_V_4.3 channels, but still with greater voltage-dependence than that in the K_V_4.1 channel (Figure 4J). Taken together, these data suggested that non-homologous key residues in the non-conserved S3b segments of K_V_4.1–4.3 channels determined their voltage-dependence phenotypes in inhibition by κ-LhTx-1 (i.e., P282 in K_V_4.1, D281 in K_V_4.2 and N278 in K_V_4.3).

### The Synergistic Role of Involved Key Residues

**FIGURE 5:**
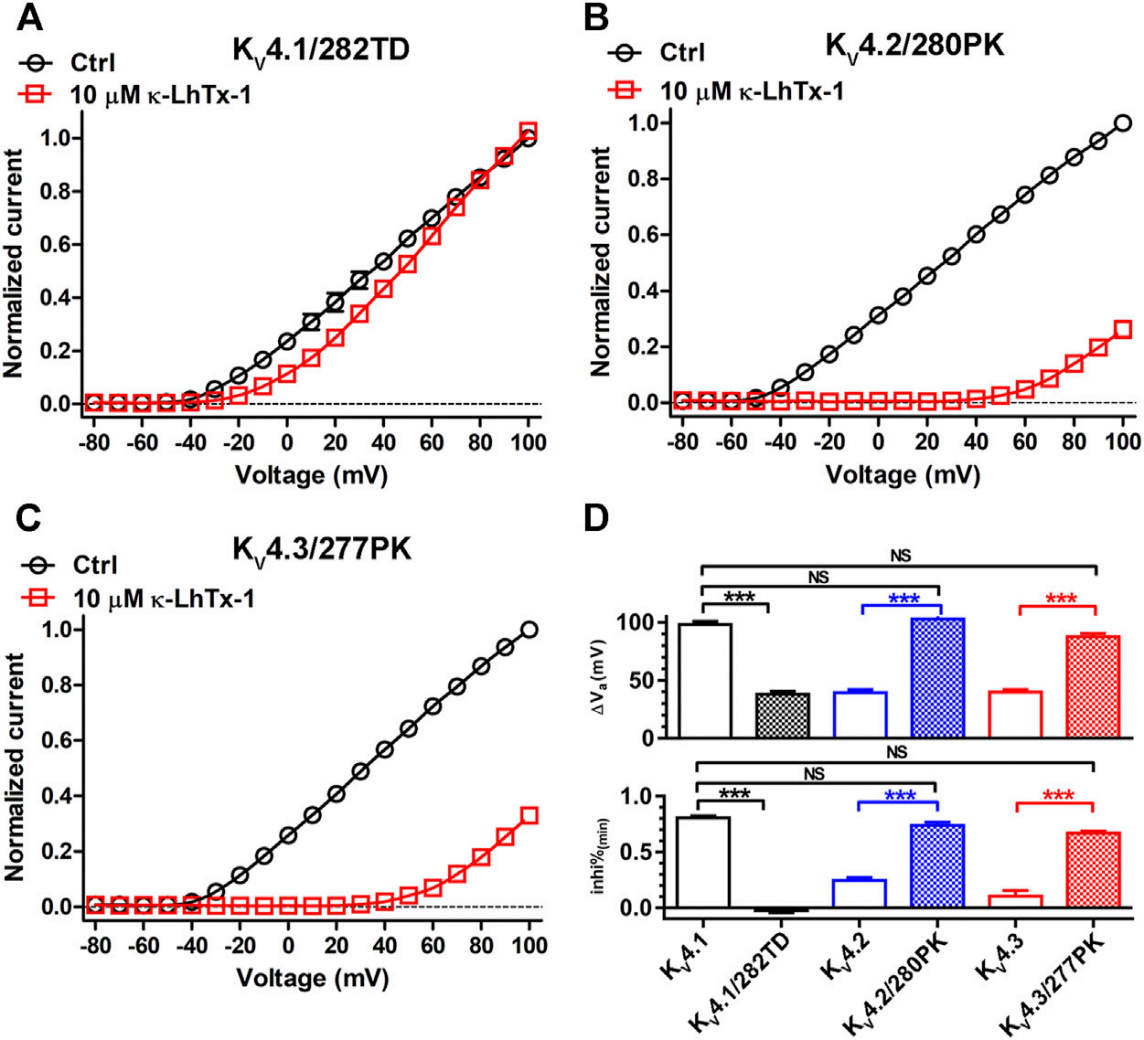
The synergistic action of involved key residues. **(A–C)**, The I-V curves of K_V_4.1/282TD **(A)**, K_V_4.2/280PK **(B)** and K_V_4.3/277PK **(C)** before and after 10 μM κ-LhTx-1 treatment (n = 5–6). Currents at all voltages were normalized to the control current (before toxin treatment) at +100 mV in each group. K_V_4.1/282TD, K_V_4.2/280PK and K_V_4.3/277PK mutants were made by mutating P282/K283 to T282/D283 in K_V_4.1, T280/D281 to P280/K281 in K_V_4.2 and T277/N278 to P277/K278 in K_V_4.3, respectively. **(D)**, The statistical diagram of the ∆V_a_ and inhi%_(min)_ values of wild-type K_V_4 channels and mutants referred in this Figure, showing K_V_4.1/282TD has the same voltage-dependence phenotype as K_V_4.2/4.3, while K_V_4.2/280PK and K_V_4.3/277PK have the same voltage-dependence phenotype as K_V_4.1, as regard to their inhibition by κ-LhTx-1 (***, *p* < 0.001; NS, not significantly different; ONE-WAY ANOVA).

Finally, we combinatorially mutated the two involved key residues in three K_V_4 channels and assessed if they two worked synergistically. Compared with the K_V_4.1/P282T channel, toxin induced an evidently smaller I-V shift in the P282T/K283D double mutation channel K_V_4.1/282TD (Figure 5A and Figure 4B). The ∆V_a_ value for K_V_4.1/282TD was significantly reduced compared with wild-type K_V_4.1 (Figure 5D, upper panel), and even smaller than that in the K_V_4.1/280VMTN chimeric channel (Supplementary Table S1). Besides, toxin’s inhibitory effect in K_V_4.1/282TD was counteracted by strong depolarizations, resulting in a inhi%_(min)_ value approaching zero (Figure 5D, lower panel). These data suggested κ-LhTx-1 inhibited K_V_4.1/282TD with stronger voltage-dependence than that in K_V_4.1/P282T and K_V_4.1/280VMTN, and even than that in K_V_4.2/K_V_4.3 channels. In line with these observations, T280P/D281K double mutations in K_V_4.2 (K_V_4.2/280PK), as well as T277P/N278K double mutations in K_V_4.3 (K_V_4.3/277PK) likely rendered toxin inhibiting channels with a further reduced voltage-dependence, compared with K_V_4.2/D281K and K_V_4.3/N278K. As shown in Figures 5B,C, toxin treatment caused a dramatic shift of the I-V curves in both channels. More importantly, the ∆V_a_ and inhi%_(min)_ values for K_V_4.2/280PK and K_V_4.3/277PK were not significantly different from those in K_V_4.1, showing that the two mutant channels fully restored the voltage-dependence phenotype of K_V_4.1. Taken together, these data suggested P282 and K283 in K_V_4.1, T280 and D281 in K_V_4.2, as well as T277 and N278 in K_V_4.3 worked synergistically in defining channel’s voltage-dependence phenotype in inhibition by κ-LhTx-1.

## Discussion

The present study has identified the peptide toxin κ-LhTx-1 from the venom of spider *Pandercetes sp* as a novel high-affinity and high-selectivity antagonist of the K_V_4 channels. κ-LhTx-1 inhibited K_V_4.2/K_V_4.3 channels with relatively higher potency than that for K_V_4.1, regardless of their extremely high sequence homology. Mutation analysis has confirmed that κ-LhTx-1 bound to the same S3b region in all three K_V_4 channels. Moreover, the action of κ-LhTx-1 on these K_V_4 channels was mostly featured by it inhibiting K_V_4.2/4.3 channels with significantly higher voltage-dependence than that of K_V_4.1, suggesting that their gating was differently modulated by the toxin. We then explored the underlying mechanism and found that the non-conserved S3b segment containing four residues in the channel is the molecular determinant, with swapping this segment between K_V_4.1 and K_V_4.3 fully exchanged their voltage-dependence phenotypes in inhibition by toxin. Interestingly, non-homologous key residues were identified in K_V_4.1 and K_V_4.2/K_V_4.3 (P282 in K_V_4.1, D281 in K_V_4.2 and N278 in K_V_4.3). Taken together, these results have revealed the structure differences in K_V_4 channels underlying their different modulations by κ-LhTx-1, which would deepen our understanding on their structure-function relationships.

The gating of K_V_4 channels could be modeled with the following scheme: C_0_⇌C_1_⇌C_2_⇌C_3_⇌C_4_⇌O, in which C, O and the subscript number represents the closed state, the open state, and the number of activated voltage sensors in each closed state, respectively (for clarity, the parameters between states transition were omitted) (Wang et al., 2004). This scheme assumes that the channel can only reach the open state with all of its four voltage sensors being activated, consequently the C_0_ to C_4_ states transition is voltage-dependent and the C_4_→O transition is an allosteric voltage-independent process. Gating modifier toxins which stabilize K_V_4 channels’ resting voltage sensor would impede its activation by voltage, increase the energy barrier for channel opening and eventually shift channel’s I-V/G-V relationships to the depolarizing direction. Actually, It’s a commonly shared mechanism for gating modifier toxins acting on various types of voltage-gated ion channels (Catterall et al., 2007). On the other hand, depolarizing voltages could partially or fully counteract toxin’s inhibition on voltage sensor, resulting in different inhibition on the currents at different voltages, defined as voltage-dependent inhibition (Phillips et al., 2005). Gating modifier toxins of K_V_4 channels, such as Ctri9557, JZTX-XII, JZTX-V and PaTx-1, all likely act in this way (Diochot et al., 1999; Yuan et al., 2007; Xie et al., 2014; Zhang et al., 2019b). It could be reasonably speculated that a larger I-V/G-V shift and less voltage-dependent inhibition would be observed in toxins stabilizing the resting voltage sensor much more stably, and vice versa. κ-LhTx-1 in the present study was also a gating modifier of K_V_4 channels, whereas it modulated the gating of K_V_4.1 and K_V_4.2/4.3 channels with significantly different voltage-dependence. Actually, the different modulations of K_V_4 channels by the same toxin isn’t without precedent. As aforementioned in the introduction section, HpTx-2 also inhibits K_V_4.1 and K_V_4.3 with different voltage-dependence. Although κ-LhTx-1 and HpTx-2 all inhibited K_V_4.1 with less voltage-dependence than that of K_V_4.3, there exist striking differences regarding their actions on the two channels, as HpTx-2 treatment shifted the G-V curve of K_V_4.1 much more less than that in K_V_4.3 while the opposite effect was observed for κ-LhTx-1. The Markov models used to illustrate the data proposed that HpTx-2 mainly affected the voltage-independent C_4_→O transition in K_V_4.1 but the voltage-dependent C_0_→C_4_ transition in K_V_4.3, which made a larger G-V shift in K_V_4.3 reasonable (DeSimone et al., 2011). However, based on our data, we proposed that κ-LhTx-1 trapped the K_V_4.1 voltage sensor in the resting state more stably than that in K_V_4.2/K_V_4.3, causing it inhibited K_V_4.1 with less voltage-dependence than the other two channels. In an other word, κ-LhTx-1 mainly impeded the voltage-dependent C_0_→C_4_ transition in all three K_V_4 channels, but with different efficiency toward them.

κ-LhTx-1 in the present study is the first reported peptide toxin from the venom of spider *Pandercetes sp* with explicitly identified activity. Given the unique action mode of κ-LhTx-1 on the K_V_4 family members, it could be used as an useful pharmacological tool to discriminate K_V_4.1 from K_V_4.2/4.3 channels. Moreover, in light of the high affinity and high selectivity of κ-LhTx-1 on K_V_4.2/4.3 channels, this toxin might represent a valuable drug lead for developing antiarrhythmics by inhibiting I_to_ currents (Antzelevitch and Patocskai, 2016; Antzelevitch et al., 2017). The inhibition of κ-LhTx-1 on K_V_4 channels in CNS might cause side-effect when considering its use in anti-arrhythmia, however, this probability could be further reduced as the ICK type toxins are expected to not cross the blood-brain barrier (BBB) freely. On the other hand, completely blocking the activity of K_V_4.2/K_V_4.3 channels might bring strong side-effect, as their mediated I_to_ currents plays fundamental role in maintaining the normal function of heart (Kuo et al., 2001). Partial inhibition of I_to_ currents is therefore a more desirable strategy (Antzelevitch and Patocskai, 2016). The feature that κ-LhTx-1 inhibited K_V_4.2/4.3 with dramatically strong voltage-dependence adds the value of using it as a more safe anti-arrhythmic drug, as it would only moderately modulate but not completely abolish the activities of K_V_4.2/4.3 channels in response to a AP (action potential)-like voltage ramp *in vivo*. Future study could be to test the effect of κ-LhTx-1 in anti-arrhythmia using cell and animal models. We have not explored the molecular determinants in κ-LhTx-1 underlying its binding with K_V_4 channels in the present study. However, sequence alignment revealed that κ-LhTx-1 and other K_V_4 active toxins including JZTx-XII, PaTx-1 and HpTx-2 share relatively high homology at their C-termini, which suggests that this segment might account for their common activity on K_V_4 channels. This speculation also needs to be experimentally checked in future studies.

## Data Availability

The raw data supporting the conclusions of this article will be made available by the authors, without undue reservation, to any qualified researcher.
