# Supplementary material for: Variation of Two S3b Residues in KV4.1–4.3 Channels Underlies Their Different Modulations by Spider Toxin κ-LhTx-1
Source: Front Pharmacol. 2021 Jun 10;12:692076. doi: 10.3389/fphar.2021.692076 (PMC8222713; doi:10.3389/fphar.2021.692076)
Supplement: Supplementary file 1 [file DataSheet1.docx]

Supplementary Information

**Variation of two S3b residues in K_V_4.1-4.3 channels underlies their different modulations by spider toxin κ-LhTx-1**

Zhen Xiao^1,#^, Piao Zhao^1,#^, Xiangyue Wu^1^, Xiangjin Kong^1^, Ruiwen Wang^1^, Songping Liang^1^, Cheng Tang^1,*^, Zhonghua Liu^1,*^

^1^The National and Local Joint Engineering Laboratory of Animal Peptide Drug Development, College of Life Sciences, Hunan Normal University, Changsha 410081, China.

^#^These authors contributed equally to this work.

^*^Correspondence: [chengtang@hunnu.edu.cn (C.T.);](mailto:chengtang@hunnu.edu.cn;) [Liuzh@hunnu.edu.cn](mailto:Liuzh@hunnu.edu.cn) (Z.L.); Tel: +86-731-8887-2556(C.T. & Z.L.)


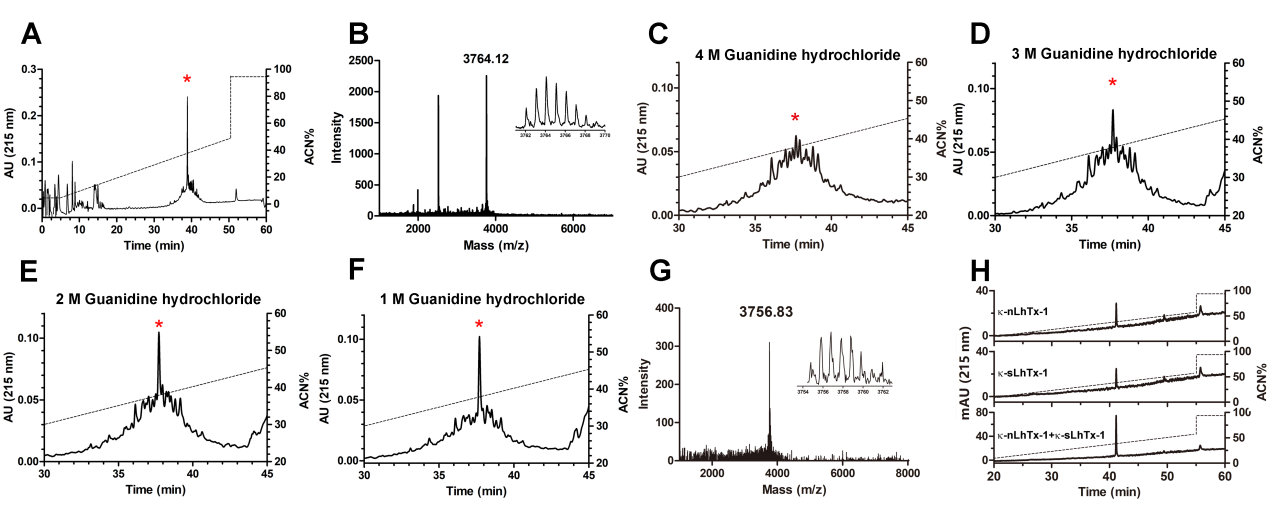


**Supplementary Figure 1.** Solid-phase synthesis and in-vitro refolding of κ-LhTx-1. (A), RP-HPLC analysis of the crude synthetic product, the red asterisk labeled peak contains the correctly-synthesized linear κ-LhTx-1. (B), MALDI-TOF MS analysis of linear κ-LhTx-1, inset shows its single isotopic MW. (C) - (F), RP-HPLC analysis showing the refolding of κ-LhTx-1 was promoted by gradually diluting the guanidine hydrochloride from 4M to 1M, the asterisk labeled peaks contain correctly-refolded toxin. (G), MALDI-TOF MS analysis of the correctly-refolded κ-LhTx-1, inset shows its single isotopic MW. (H), Co-elution analysis of of native and synthesized κ-LhTx-1 (referred to as κ-nLhTx-1 and κ-sLhTx-1, respectively).


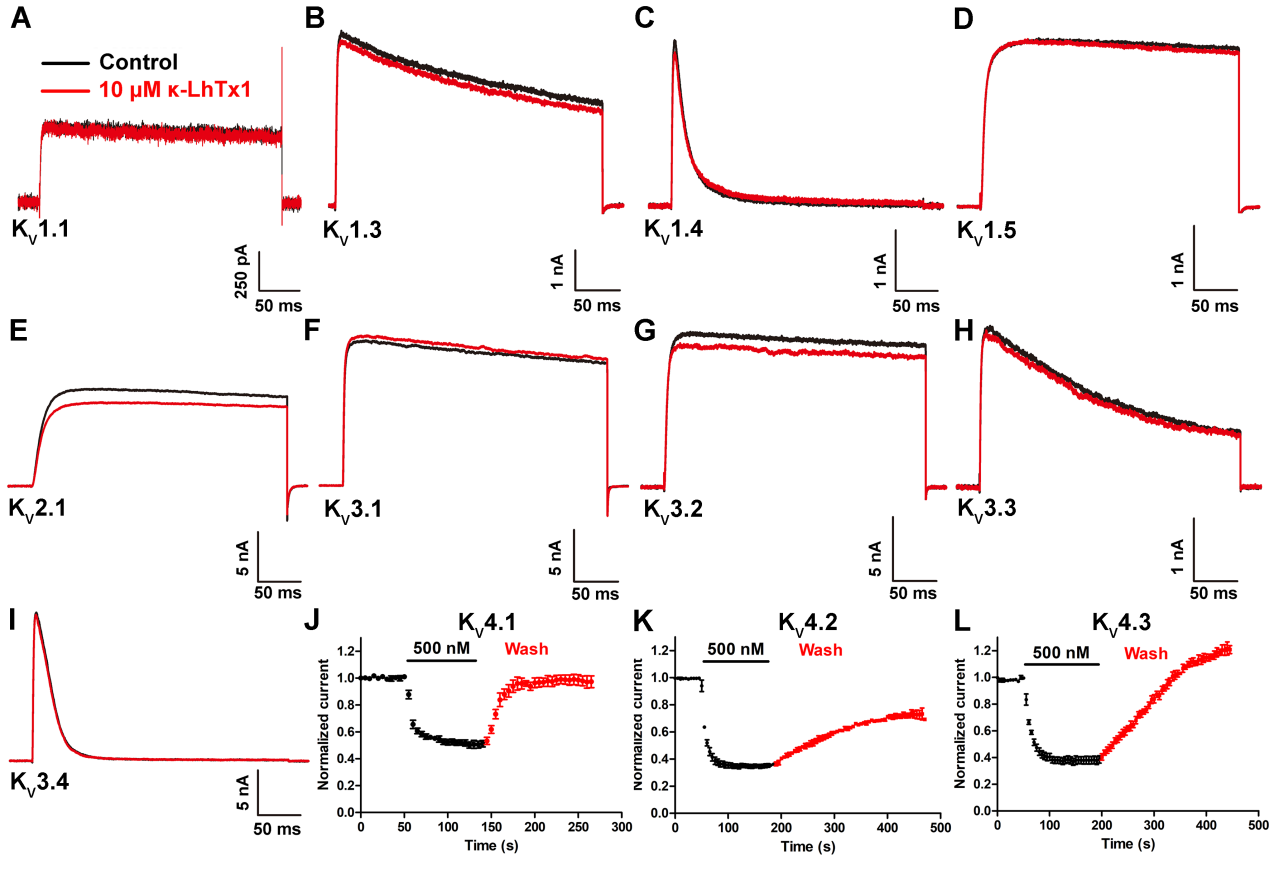


**Supplementary Figure 2.** κ-LhTx-1 selectively and reversibly blocks K_V_4 channels. (A) - (I), 10 μM κ-LhTx-1 does not remarkably inhibit the currents mediated by K_V_1.1, K_V_1.3-1.5, K_V_2.1 and K_V_3.1-3.4 channels (n = 5). (J) - (L), Reversible inhibition of K_V_4.1 (J), K_V_4.2(K) and K_V_4.3(L) by κ-LhTx-1.

| **Channel** | **Steady state activation (mV)** | | | | | | | **Steady state inactivation (mV)** | | | | | | |
| --- | --- | --- | --- | --- | --- | --- | --- | --- | --- | --- | --- | --- | --- | --- |
|  | **Control** | | | **10 μM κ-LhTx-1** | | | | **control** | | | **10 μM κ-LhTx-1** | | | |
|  | **V_a_** | **k** | **n** | **V_a_** | **k** | **n** | **∆V_a_** | **V_h_** | **k** | **n** | **V_h_** | **k** | **n** | **∆V_h_** |
| **K_V_4.1** | -3.09 ± 1.84 | 20.30 ± 0.95 | 6 | 95.20 ± 1.68 | 16.14 ± 1.78 | 5 | 98.28 ± 2.66 | -61.75 ± 0.56 | 5.50 ± 0.12 | 5 | -32.48 ± 3.44 | 22.88 ± 0.19 | 5 | 29.27 ± 2.05 |
| **K_V_4.1/P282T** | -0.03 ± 1.17 | 19.82 ± 0.57 | 5 | 52.55 ± 1.38 | 18.09 ± 0.43 | 6 | 52.58 ± 1.86 | -55.87 ± 2.11 | 4.98 ± 0.16 | 7 | -29.12 ± 1.84 | 10.73 ± 0.41 | 6 | 26.75 ± 2.85 |
| **K_V_4.1/K283D** | -11.72 ± 2.54 | 17.91 ± 0.77 | 8 | 91.34 ± 3.47 | 17.49 ± 1.57 | 5 | 103.10 ± 4.22 | -61.13 ± 1.43 | 7.49 ± 0.50 | 8 | -36.39 ± 3.03 | 19.23 ± 1.48 | 5 | 24.74 ± 3.63 |
| **K_V_4.1/282TD** | -8.82 ± 2.37 | 21.56 ± 1.42 | 5 | 29.00 ± 0.52 | 25.50 ± 0.51 | 5 | 37.82 ± 2.83 | -59.60 ± 1.80 | 7.21 ± 0.42 | 5 | -56.22 ± 2.80 | 5.95 ± 0.32 | 5 | 3.38 ± 3.15 |
| **K_V_4.1/280VMTN** | -2.83 ± 1.05 | 21.12 ± 0.35 | 8 | 58.61 ± 1.63 | 19.00 ± 0.81 | 5 | 61.44 ± 1.98 | -58.06 ± 1.00 | 7.25 ± 0.23 | 8 | -44.55 ± 2.07 | 9.86 ± 1.33 | 5 | 13.52 ± 2.24 |
| **K_V_4.2** | -9.28 ± 1.78 | 22.36 ± 0.38 | 6 | 30.24 ± 1.82 | 21.99 ± 0.42 | 8 | 39.52 ± 2.55 | -63.43 ± 1.58 | 5.03 ± 0.43 | 6 | -69.67 ± 1.87 | 7.22 ± 0.26 | 6 | 6.25 ± 2.45 |
| **K_V_4.2/T282P** | 19.67 ± 3.90 | 30.60 ± 1.66 | 5 | 16.93 ± 2.90 | 25.61 ± 0.68 | 5 | 2.742 ± 5.10 | -67.11 ± 2.19 | 6.15 ± 0.24 | 13 | -71.72 ± 3.71 | 5.86 ± 0.21 | 5 | 4.61 ± 4.45 |
| **K_V_4.2/D281K** | -6.14 ± 1.93 | 2.24 ± 0.55 | 10 | 61.96 ± 1.96 | 18.73 ± 0.70 | 6 | 68.10 ± 2.92 | -60.67 ± 1.52 | 6.25 ± 0.67 | 6 | -27.43 ± 2.53 | 15.24 ± 1.42 | 6 | 33.23 ± 2.95 |
| **K_V_4.2/280PK** | -20.61 ± 2.75 | 23.55 ± 1.16 | 6 | 82.17 ± 1.67 | 13.22 ± 1.15 | 6 | 102.8 ± 3.22 | -53.59 ± 2.36 | 5.99 ± 0.38 | 5 | -9.93 ± 2.94 | 11.46 ± 1.10 | 5 | 43.66 ± 3.77 |
| **K_V_4.3** | 6.97 ± 1.52 | 25.37 ± 2.19 | 13 | 46.86 ± 1.52 | 20.45 ± 0.30 | 10 | 39.88 ± 2.19 | -45.69 ± 2.18 | 5.39 ± 0.18 | 5 | -30.87 ± 2.23 | 6.21 ± 0.43 | 7 | 14.83 ± 3.34 |
| **K_V_4.3/N278K** | 8.65 ± 1.64 | 23.21 ± 1.83 | 12 | 68.62 ± 1.73 | 18.03 ± 0.87 | 6 | 59.97 ± 2.64 | -47.25 ± 0.98 | 7.12 ± 0.27 | 11 | -17.01 ± 1.57 | 10.75 ± 0.63 | 5 | 30.24 ± 1.80 |
| **K_V_4.3/T277P** | 8.71 ± 2.07 | 25.00 ± 0.611 | 10 | 49.17 ± 0.88 | 22.21 ± 1.64 | 6 | 40.47 ± 2.79 | -47.09 ± 1.32 | 5.86 ± 0.13 | 11 | -35.46 ± 1.68 | 8.50 ± 0.42 | 6 | 11.63 ± 2.17 |
| **K_V_4.3-TN/PK** | -5.47 ± 1.94 | 22.00 ± 0.81 | 7 | 81.96 ± 2.27 | 15.09 ± 1.14 | 5 | 87.43 ± 2.99 | -51.72 ± 1.94 | 6.03 ± 0.15 | 7 | -25.43 ± 2.92 | 15.31 ± 2.02 | 5 | 26.29 ± 3.52 |
| **K_V_4.3/275FVPK** | -1.31 ± 1.45 | 22.24 ± 0.68 | 7 | 93.50 ± 2.00 | 17.66 ± 1.15 | 5 | 94.81 ± 2.45 | -52.62 ± 2.69 | 9.81 ± 0.39 | 10 | -9.28 ± 5.57 | 16.78 ± 3.11 | 5 | 43.34 ± 5.46 |

**Supplementary Table 1.** Summary for κ-LhTx-1 treatment affecting the gating parameters (V_a_, V_h_ and K) of channels used in this research.
